# Coordinated Destruction of Cellular Messages in Translation Complexes by the Gammaherpesvirus Host Shutoff Factor and the Mammalian Exonuclease Xrn1

**DOI:** 10.1371/journal.ppat.1002339

**Published:** 2011-10-27

**Authors:** Sergio Covarrubias, Marta M. Gaglia, G. Renuka Kumar, Wesley Wong, Andrew O. Jackson, Britt A. Glaunsinger

**Affiliations:** 1 Division of Infectious Diseases and Immunity, School of Public Health, University of California Berkeley, Berkeley, California, United States of America; 2 Department of Plant and Microbial Biology, University of California Berkeley, Berkeley, California, United States of America; University of Florida, United States of America

## Abstract

Several viruses encode factors that promote host mRNA degradation to silence gene expression. It is unclear, however, whether cellular mRNA turnover pathways are engaged to assist in this process. In Kaposi's sarcoma-associated herpesvirus this phenotype is enacted by the host shutoff factor SOX. Here we show that SOX-induced mRNA turnover is a two-step process, in which mRNAs are first cleaved internally by SOX itself then degraded by the cellular exonuclease Xrn1. SOX therefore bypasses the regulatory steps of deadenylation and decapping normally required for Xrn1 activation. SOX is likely recruited to translating mRNAs, as it cosediments with translation initiation complexes and depletes polysomes. Cleaved mRNA intermediates accumulate in the 40S fraction, indicating that recognition occurs at an early stage of translation. This is the first example of a viral protein commandeering cellular mRNA turnover pathways to destroy host mRNAs, and suggests that Xrn1 is poised to deplete messages undergoing translation in mammalian cells.

## Author Summary

Viruses use a number of strategies to commandeer host machinery and create an optimal environment for their replication. One strategy employed by oncogenic gammaherpesviruses such as Kaposi's sarcoma-associated herpesvirus (KSHV) is to block cellular gene expression through extensive destruction of mRNAs. A single viral protein called SOX is sufficient to drive this phenotype, but the mechanism by which it does so has remained unclear. Here we show that host mRNA destruction is the result of the coordinated action of SOX and a cellular RNA degrading enzyme, Xrn1. By cleaving mRNAs internally, SOX recruits the activity of Xrn1 while bypassing the regulatory mechanisms that normally prevent this enzyme from prematurely degrading mRNAs. We also find that SOX co-sediments with translation complexes, and specifically targets mRNAs for cleavage at an early stage of translation. We hypothesize this allows the virus to selectively target mRNAs, thereby liberating host gene expression machinery. Collectively, these findings describe a novel interplay between the gammaherpesvirus SOX protein and cellular degradation machinery, and shed light on how a single viral component can hijack cellular machinery to efficiently destroy messages.

## Introduction

Tight control of gene expression is achieved not only at the level of transcription, but also by modulating post-transcriptional events such as mRNA turnover. Indeed, recent studies show that changes in mRNA stability account for as much as 40% to 60% of the changes in steady-state mRNA levels in basic cellular processes such as signaling pathways [1], stress responses [2] and cell differentiation [3]. A core set of conserved enzymes is responsible for both basal and regulated mRNA degradation in eukaryotes, including two potent exoribonucleases, the 5′-3′ exonuclease Xrn1 and a 3′-5′ exonucleolytic complex called the exosome (reviewed in [4], [5]). The activity of these exonucleases on eukaryotic mRNAs is kept in check by the presence of a 5′ 7-methylguanosine cap and a 3′ poly(A) tail, which prevent access to the message body. Poly(A) removal by one of the cellular deadenylases is likely the rate limiting step of basal mRNA degradation [6] and is in turn required for message decapping [7] by the Dcp2 enzyme in complex with the activator Dcp1A and other cofactors [8], [9]. Endonucleases are also emerging as important contributors to eukaryotic mRNA decay, particularly in the context of quality control pathways or other situations requiring rapid inactivation of select messages [10].

Because of the central role of mRNA degradation in gene expression regulation, viruses presumably interact with cellular RNA degradation machinery in the process of taking control of the cell for their own replication. Viruses often interface with degradation pathways to prevent turnover of their genomic or messenger RNAs [11], [12], and can downregulate specific messages using both viral and cellular microRNAs (miRNAs), often to modulate immune evasion or other steps in their lifecycle [13]. Some viruses can even co-opt core mRNA degradation components for their own purposes, as flaviviruses do to generate noncoding subgenomic viral RNAs [14]. However, no example has been found of viruses that use the mRNA turnover machinery to broadly target cellular messages for destruction. Several virus-encoded factors can cause widespread degradation of cellular mRNAs and block host gene expression (host shutoff), but it is unclear what role host pathways play in this context [15], [16], [17], [18].

One of the viruses causing extensive host mRNA degradation is Kaposi's sarcoma-associated herpesvirus (KSHV), the etiologic agent of Kaposi's sarcoma, primary effusion lymphoma, and B-cell type multicentric Castleman's disease [19], [20], [21]. During lytic infection with KSHV or other human and murine gammaherpesviruses, expression of the viral protein SOX (ORF37; BGLF5 in Epstein-Barr virus) induces degradation of the majority of the cellular messenger RNAs [15], [18], [22], [23], [24]. This depletion of cytoplasmic mRNAs leads to nuclear relocalization of poly(A) binding protein (PABPC), which subsequently drives mRNA hyperadenylation in the nucleus and a concomitant mRNA export block [25], [26], [27]. Thus, SOX activity in the cytoplasm triggers a cascade of events that further restricts gene expression. SOX belongs to a family of DNA alkaline exonucleases (DNases) that are conserved in all herpesviral subfamilies and have roles in processing and maturation of the newly replicated viral DNA genomes. *In vitro*, several herpesviral SOX homologs exhibit robust 5′-3′ DNase activity and weaker exonuclease activity towards RNA substrates [28], [29], [30], [31], [32]. It is notable, however, that only the homologs from viruses of the gammaherpesvirus subfamily can promote RNA turnover in cells, and in these proteins the DNase and host shutoff activities can be genetically separated [15], [33]. It is therefore unclear to what extent the *in vitro* RNase activity contributes to the host shutoff activity in cells, and what cellular cofactors may participate in the specific targeting and efficient destruction of mRNAs.

Here, we show that the mechanism of KSHV-induced mRNA turnover involves the coordinated activities of SOX and the cellular 5′-3′ ribonuclease Xrn1. Unlike canonical cellular mRNA decay, in which Xrn1 generally gains access to mRNAs only after deadenylation and decapping, SOX generates substrates for Xrn1 that have not undergone these rate-limiting events. Our data suggest that this occurs via a SOX-induced site-specific endonucleolytic cleavage on each mRNA, thus providing an accessible 5′ end for Xrn1-mediated degradation of the mRNA body. Furthermore, we show that SOX co-sediments with 40S translation initiation complexes, and causes mRNA cleavage at an early stage of translation. SOX specifically targets polymerase (Pol) II-generated mRNAs, but not RNAs transcribed by Pol I or Pol III. This leads to a global depletion of cellular mRNAs in polysomes, and explains the preferential targeting of messenger RNAs during host shutoff. Our data suggest a model in which the virus co-opts host mRNA decay pathways to rapidly liberate cellular translation machinery, presumably to create an optimal host environment for viral gene expression and replication.

## Results

### Xrn1 participates in SOX-mediated degradation of cellular RNAs

**Figure 1:**
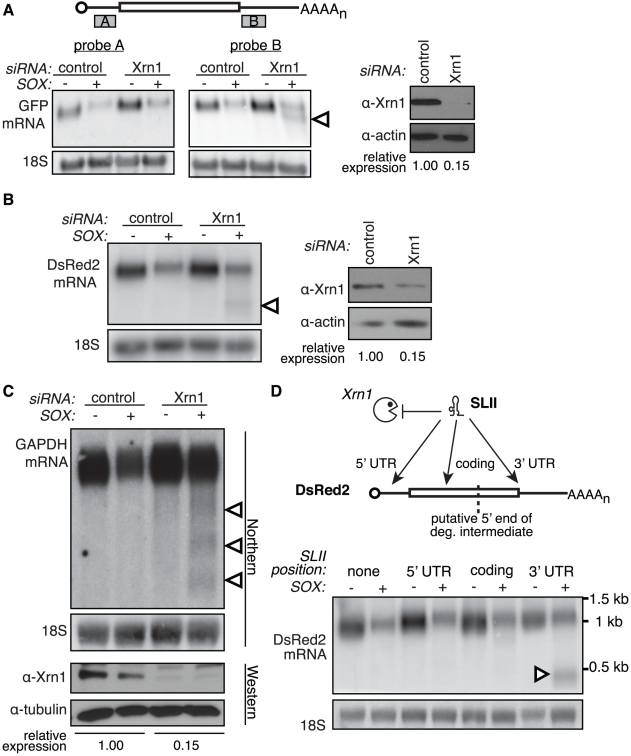
Xrn1 is required for the removal of a 3′ intermediate during SOX-mediated mRNA degradation. (A–B) 293T cells were treated with control or Xrn1 siRNAs, then transfected with the indicated reporters +/− SOX. The level of Xrn1 protein depletion is shown on the right, with actin as a loading control. (A) Diagram depicts location of probes on the GFP message. Northern blots using probes against either the 5′ (probe A; *left panel*) or 3′ (probe B; *right panel*) UTR of the GFP mRNA reporter or 18S. (B) Northern blots using probes against the 3′ UTR of the DsRed2 mRNA reporter, or 18S (*left panels*). (C) 293T cells were treated with control or Xrn1 shRNAs, then transfected with GFP (−) or GFP-SOX (+). Cells were then selected for GFP fluorescence by FACS before RNA and protein collection. RNA was Northern blotted with probes to the 3′ end of the GAPDH coding region, or to 18S. The level of Xrn1 knockdown was assessed by Western blot, using tubulin as a loading control. In panels A, B and C, arrowheads denote degradation intermediates. (D) A flaviviral Xrn1-blocking element (SLII) was inserted at different positions within the DsRed2 mRNA. RNA from 293T cells transfected with the indicated reporter +/− SOX was Northern blotted using a probe directed against the 3′ UTR of the DsRed2 reporter, or 18S. The full-length mRNA is ∼1.2 kb, and the expected size of the fragment protected from Xrn1 degradation by SLII within the 5′ UTR is ∼1.1 kb, within the coding region is ∼900 bp, and within the 3′ UTR is ∼500 bp. Arrowhead denotes protected fragment.

The KSHV protein SOX and its homologs in other gammaherpesviruses are potent inducers of cellular mRNA degradation. While they have also recently been shown to exhibit RNase activity *in vitro*, mutational analyses indicated that this activity cannot fully account for SOX-induced mRNA degradation in cells [28]. We therefore hypothesized that SOX co-opts cellular mRNA degradation machinery to enact host transcriptome destruction. We reasoned that the 5′-3′ exoribonuclease Xrn1 would be a likely candidate co-factor, as it plays a major role in basal mRNA turnover in the cytoplasm. To test the involvement of Xrn1 in SOX-mediated mRNA degradation, we monitored SOX-induced turnover of a GFP reporter mRNA in 293T cells upon transfection of Xrn1-specific versus control small interfering RNAs (siRNAs). Unexpectedly, depletion of Xrn1 did not protect full-length GFP mRNA, but it resulted instead in accumulation of a shorter GFP RNA intermediate (Figure 1A). The intermediate could be specifically detected by Northern blotting with probes against the 3′ but not the 5′ end of the mRNA (Figure 1A), indicating a 5′ end truncation. The reliance on Xrn1 to assist in the degradation of mRNA in SOX-expressing cells was also observed for three additional reporter mRNAs, including DsRed2 (Figure 1B and S1A-B) and for the endogenous cellular transcript glyceraldehyde 3-phosphate dehydrogenase (GAPDH, Figure 1C). Notably, although the full-length GFP and DsRed2 reporter mRNAs are of similar size (1.2–1.5 kb), the lengths of their degradation intermediates were different: the GFP fragment was approximately 1.1 kb whereas the DsRed2 fragment was ∼600 bp (data not shown). In addition, the GAPDH mRNA generated multiple cleavage intermediates. This indicates that the generation of the intermediates is not controlled by a positional cue, such as distance from the mRNA ends.

We further confirmed that Xrn1 is involved in SOX-induced RNA degradation in an siRNA-independent manner by use of a flaviviral structural element (SLII) that can block 5′-3′ RNA degradation by Xrn1 [14]. In SOX expressing cells, insertion of the SLII element within the 3′ UTR of the DsRed2 reporter resulted in the appearance of a protected fragment of a size consistent with the portion of the mRNA downstream of the SLII (Figure 1D). In contrast, RNA fragments were not observed upon insertion of the SLII in the DsRed2 coding region upstream of the predicted 5′ end of the intermediate or in the 5′ UTR. This agrees with the observation that the 5′ portion of the mRNA is removed via an Xrn1-independent mechanism. Results consistent with this interpretation were also obtained for the GFP and β-globin reporters (Figure S1C–E). Collectively, our data suggest that SOX-mediated RNA depletion is a two-step process: an initiating event that removes a portion of the 5′ end of the mRNA, which is then followed by exonucleolytic degradation of the remaining fragment by the host Xrn1 enzyme.

### SOX-induced mRNA degradation bypasses the requirement for deadenylation and decapping

**Figure 2 ppat-1002339-g002:**
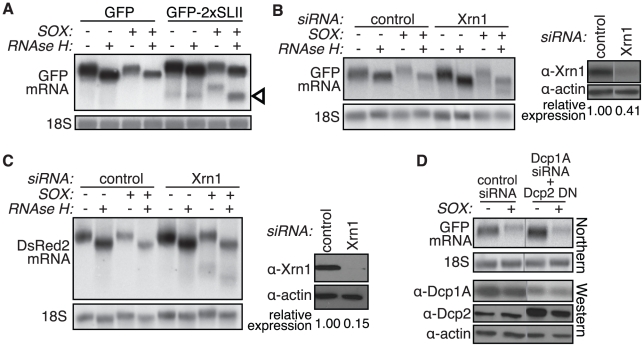
The 3′ degradation intermediate retains its poly(A) tail. (A) 293T cells were transfected with GFP or a modified GFP reporter containing two copies of the flaviviral Xrn1-blocking element SLII within the GFP coding region, +/− SOX. A fraction of the RNA was treated with oligo(dT) and RNAse H to remove the poly(A) tail prior to Northern blotting with a GFP 3′ UTR or an 18S probe. Arrowhead denotes protected fragments. (B–C) 293T cells were transfected with control or Xrn1 siRNAs, followed by GFP (B) or DsRed2 (C) reporters +/− SOX. A fraction of the RNA was treated with oligo(dT) and RNAse H to remove the poly(A) tail prior to Northern blotting with a GFP 3′ UTR or an 18S probe. Xrn1 protein levels were assessed by Western blot (*right panels* in B and C). (D) 293T cells were treated with control siRNAs or siRNAs against the decapping complex protein Dcp1A. They were then transfected with GFP +/− SOX and/or the Dcp2 dominant negative mutant E148Q (Dcp2 DN). RNA was Northern blotted using a GFP 3′ UTR or an 18S probe. Western blots show the level of Dcp1A knockdown and Dcp2 DN overexpression. Actin serves as a loading control and grey lines indicate where intervening lanes have been cropped out. See also Figure S2.

In mammalian cells, sequential removal of the protective 3′ poly(A) tail and 5′ cap is generally required for Xrn1 to gain access to the RNA, as it can only degrade RNAs with a free 5′ monophosphate. Moreover, deadenylation is thought to be the rate-limiting step regulating mRNA degradation. Several results, however, indicate that SOX promotes mRNA degradation by Xrn1 while bypassing the requirement for deadenylation. Whereas the effects of a single copy of the Xrn1-blocking SLII element were only apparent in cells transfected with SOX (as these undergo enhanced mRNA turnover) (Figure 1D and S1D-E), insertion of two copies of the SLII (GFP-2xSLII, Figure S2A) led to a stronger Xrn1 block that can be visualized during basal GFP mRNA turnover even in cells lacking SOX (Figure 2A). In the absence of SOX, *in vitro* removal of the poly(A) tails by incubation with oligo(dT) and RNase H had little effect on the mobility of the degradation fragment resulting from the GFP-2xSLII construct, confirming that this fragment was generated after deadenylation. In contrast, the fragment produced in SOX-expressing cells was significantly larger prior to RNase H treatment, indicating that the cleaved fragment retained its poly(A) tail (Figure 2A).

Similarly, we used RNAse H assays to evaluate the polyadenylation status of the SOX degradation intermediate present in Xrn1-depleted cells. Poly(A) tail removal decreased the mobility of both the full-length GFP and DsRed2 mRNAs, as well as the Xrn1-targeted degradation intermediates (Figure 2B and 2C). In addition, we were able to purify the GFP intermediate after poly(A) selection of the mRNA over oligo(dT) coupled beads (data not shown). Together, these data indicate that SOX can bypass the main regulatory step of normal mRNA degradation and render RNAs directly accessible to Xrn1.

Given that deadenylation generally precedes mRNA cap removal, we predicted that mRNA degradation in SOX-expressing cells would occur in a decapping-independent manner. Consistent with our hypothesis, we observed SOX-induced mRNA turnover in cells overexpressing a dominant negative mutant of the Dcp2 decapping enzyme, Dcp2 E148Q (J. Lykke-Andersen, personal communication; [34]), that had also been subjected to siRNA-mediated depletion of the decapping co-activator Dcp1A (Figure 2D and S2C). Although at least one additional decapping enzyme exists in mammalian cells [35], overexpression of the Dcp2 mutant was sufficient to reduce basal mRNA turnover of the GFP-2xSLII reporter, consistent with an inhibition of decapping activity (Figure S2B). We also depleted levels of the hRrp41 subunit of the 3′-5′ exosome and saw no difference between control and exosome siRNA-treated cells in our assay, in agreement with previous observations [25] (Figure S2D). Neither 5′ nor 3′ degradation fragments were detected upon hRrp41 knockdown (Figure S2E). A caveat with siRNA knockdown experiments in general is that negative results could be due to insufficient depletion of the protein. However, taken together our data suggest that the viral SOX protein interfaces specifically with the Xrn1 enzyme to accomplish host shutoff. In addition, these results further argue that removal of the 5′ portion of the RNA occurs independently of the canonical 5′-3′ decay pathway.

### A specific element directs the initial cleavage

**Figure 3 ppat-1002339-g003:**
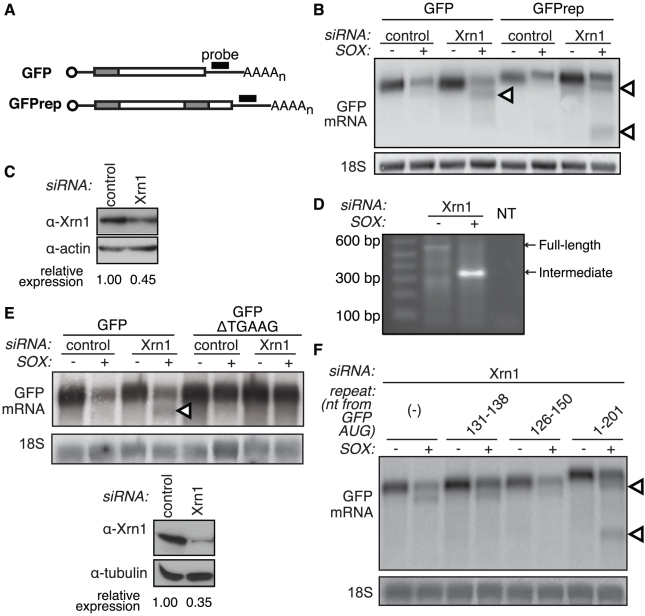
A specific element in the GFP coding region is sufficient for production of degradation intermediates by SOX. (A) The modified GFP mRNA (GFPrep) contains an internal repeat of the first 201 basepairs of the GFP coding region (grey shaded area). (B) 293T cells were treated with control or Xrn1 siRNAs, then transfected with GFP or GFPrep +/− SOX. RNA was Northern blotted with a GFP 3′ UTR or an 18S probe. Arrowheads denote degradation intermediates. (C) Western blot shows the level of Xrn1 depletion in the experiment depicted in panel B, with actin as a loading control. (D) 5′ RACE was carried out on total RNA from 293T cells transfected with Xrn1 siRNAs and a GFP expression vector in the presence or absence of SOX. An internal GFP primer and a primer to the adapter ligated to the 5′ end of the RNAs were used to amplify GFP in each sample. *Full length* refers to the band corresponding to the intact GFP transcript, *intermediate* refers to the ∼300 bp band preferentially amplified in the lysate from SOX-expressing cells, and *NT* denotes the no template control. (E) A conserved TGAAG site located just upstream of the cleavage site was deleted from GFP to create GFP-ΔTGAAG. 293T cells were treated with control or Xrn1 siRNAs, then transfected with GFP or GFP ΔTGAAG in the presence or absence of SOX. RNA was Northern blotted with a GFP 3′ UTR or 18S probe (*upper panels*). Arrowheads denote degradation intermediates. Western blot *(lower panels)* shows the level of Xrn1 depletion in experiments depicted in panel E and F, with tubulin as a loading control. (F) Modified GFP reporters bearing an internal duplication of sequences around the GFP cleavage site were transfected in 293T cells treated with Xrn1 siRNAs +/− SOX. The inserted nucleotides are numbered based on their position relative to the GFP start codon. GFPrep (nt 1–201) is included as a positive control. RNA was Northern blotted with a GFP 3′ UTR or 18S probe. Arrowheads denote degradation intermediates.

One way that mRNAs can be made accessible to Xrn1 without prior deadenylation or decapping is by internal endonucleolytic cleavage, a mechanism used by host quality control pathways to rapidly eliminate flawed mRNAs. Indeed, the defined length of the intermediates that we observe following Xrn1 depletion is strikingly reminiscent of the Smg6-cleaved intermediates seen during nonsense mediated decay (NMD) [36], [37]. A defined-length intermediate also suggests that cleavage is directed to a specific location or sequence within the mRNA. The different size of the intermediate observed in GFP and DsRed2 (compare Figures 1A and 1B) further indicated that a specific element, rather than positional information, is likely to direct the truncation. To test whether there is a specific sequence that leads to an endonucleolytic cut in the presence of SOX, we constructed a modified GFP reporter that contained an internal in-frame repeat of a 201 nt region encompassing the predicted cleavage site (Figure 3A, GFPrep). If this region contained an element targeted by an endonuclease, we expected that in the presence of SOX, GFPrep would give rise to two intermediates, assuming that the two cleavage events were equally likely and mutually exclusive. Indeed, we found that the GFPrep reporter generated two intermediates of sizes corresponding to two independent cleavage events (Figure 3B, arrowheads). These data suggest that an element within the initial 201 nucleotides of the GFP coding region directs an endonucleolytic cleavage in SOX-expressing cells.

To identify sequences involved in directing the internal cleavage, we used 5′ rapid amplification of cDNA ends (RACE) to map the 5′ end of the degradation intermediates found in GFP, DsRed2 and β-globin. In all cases our RACE results confirmed what we had previously seen with the Northern blot analysis, in that a single predominant intermediate was amplified in Xrn1-depleted cells in a SOX-dependent manner (Figure 3D and data not shown). The majority of the sequences within this band terminated at a single nucleotide or within few nucleotides of each other (Figure S3). Analysis of the sequences surrounding the cleavage site for each mRNA revealed a conserved stretch of five bases (TGAAG) just upstream of the cleavage site. Deletion of the TGAAG element from the GFP construct abolished production of the cleavage fragment, confirming its role in SOX targeting (Figure 3E). However, insertion of this sequence alone (nt 131–138) or of a 25-nucleotide stretch (nt 126–150) surrounding the GFP cleavage site was not sufficient to elicit generation of a second fragment (Figure 3F). We therefore hypothesize that the TGAAG sequence is an essential component of a larger element, perhaps structural, involved in directing endonucleolytic cleavage of mRNAs in SOX-expressing cells.

### The catalytic residues of SOX are required for host shutoff

**Figure 4:**
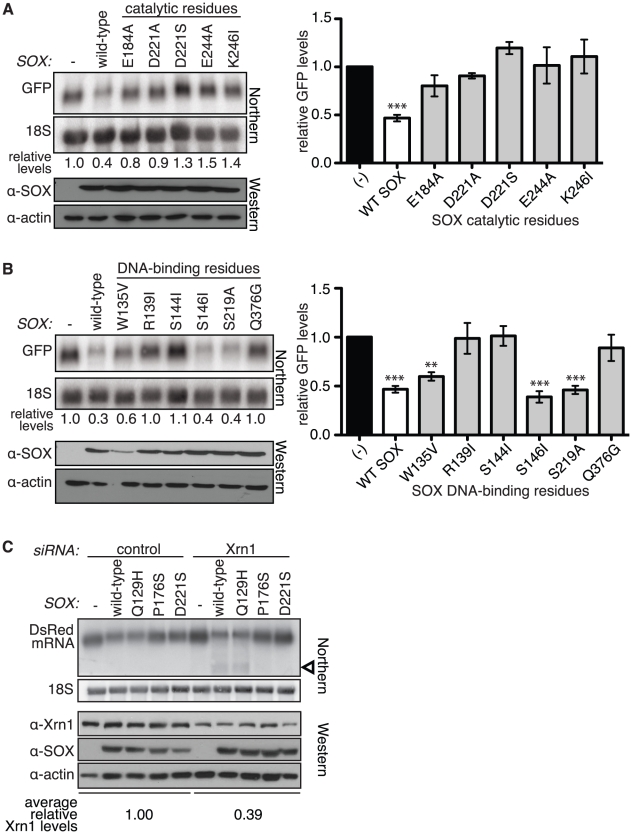
The catalytic residues of SOX are required for mRNA turnover and generation of the degradation intermediate. (A–B) Wild-type (WT) SOX or SOX putative catalytic (A) or DNA binding (B) residue mutants were transfected into 293T cells with a GFP reporter. RNA was Northern blotted using a 5′ GFP or 18S probe, and protein lysates were Western blotted for SOX and actin (as a loading control). Graphs depicting mean relative GFP levels ± s.e.m. from >3 experiments in cells transfected with the various mutants are also shown (*right*). **p<0.01, ***p<0.001, One-way ANOVA followed by Dunnett's test versus (−). (C) 293T cells were transfected with control or Xrn1 siRNAs, followed by a DsRed2 reporter +/− WT SOX or the indicated SOX mutant. Northern blots were performed using a probe directed against the 3′ UTR of the DsRed2 mRNA or 18S (*upper panels*), and Western blots show expression of the SOX mutants and the level of Xrn1 depletion (*lower panels*). Arrowhead denotes degradation intermediates.

*In vitro,* several herpesviral homologs of SOX exhibit weak DNA endonuclease activity as well as RNase activity on linear RNA substrates [28], [29], [30], [38], [39], [40]. Thus, it is possible that SOX itself carries out the initial truncation of mRNAs in cells. Recently, the structure of SOX and its homolog BGLF5 in Epstein-Barr virus were solved, leading to the identification of catalytic residues responsible for the DNase activity of these proteins, as well as putative DNA binding residues [28], [30], [41]. Mutation of one of the catalytic residues in BGLF5 (D203S) was shown to abolish all *in vitro* nuclease activity without misfolding the protein [30]. To test whether the SOX catalytic core is required for host shutoff *in vivo*, we generated individual SOX mutants for each of the catalytic residues identified in the crystal structures: E184A, D221A or D221S (equivalent to D203S in BGLF5), E244A and K246I. Indeed, mutation of any one of the catalytic residues abolished the ability of SOX to deplete GFP mRNA in cells (Figure 4A). As a control, we mutated the putative DNA-binding residues (W135V, R139I, S144I, S146I, S219A, Q376G), which are also located in the active site cleft (Figure S4C), and found that only residues R139, S144 and Q376 were required for host shutoff (Figure 4B). This suggests that SOX may bind RNA and DNA using partially overlapping residues. In contrast with our host shutoff data, all the catalytic and putative DNA binding residues tested were required for *in vitro* DNase activity (Figure S4A and S4B). Thus, our mutational analysis suggests that the catalytic activity of SOX- presumably the RNase activity- is required for host shutoff.

We next tested directly whether the RNA turnover activity of SOX was responsible for the generation of the degradation intermediate. We compared production of the degradation intermediate when expressing a SOX mutant lacking only DNase activity associated with viral genome processing (Q129H) [33], only the mRNA turnover activity (P176S) [33], or a catalytic mutant lacking both activities (D221S) (Figure 4C). The degradation intermediate was not produced in either of the two mutants lacking RNA turnover activity, but was readily detectable in cells expressing Q129H, which selectively lacks DNase function. These results indicate that generation of the intermediate by SOX is closely linked to its ability to degrade mRNAs, consistent with the two-step model for SOX-mediated mRNA degradation.

### SOX depletes polysomes and co-sediments with 40S subunits

We next sought to determine how SOX targets cytoplasmic mRNAs for the initial cleavage event. *In vitro*, SOX exhibits relatively weak affinity for RNA (*K*_d_ of 87 µM), suggesting that in cells it is recruited to mRNAs via associations with host factors [28]. In cellular quality control pathways such as NMD, translation is required for error recognition and the primary endonucleolytic cleavage [42], [43]. In addition, it has recently been reported that the vast majority of mRNAs in the cytoplasm are polysome-associated [44], suggesting that targeting mRNAs engaged in translation would be an efficient mechanism to clear host messages during host shutoff.

**Figure 5:**
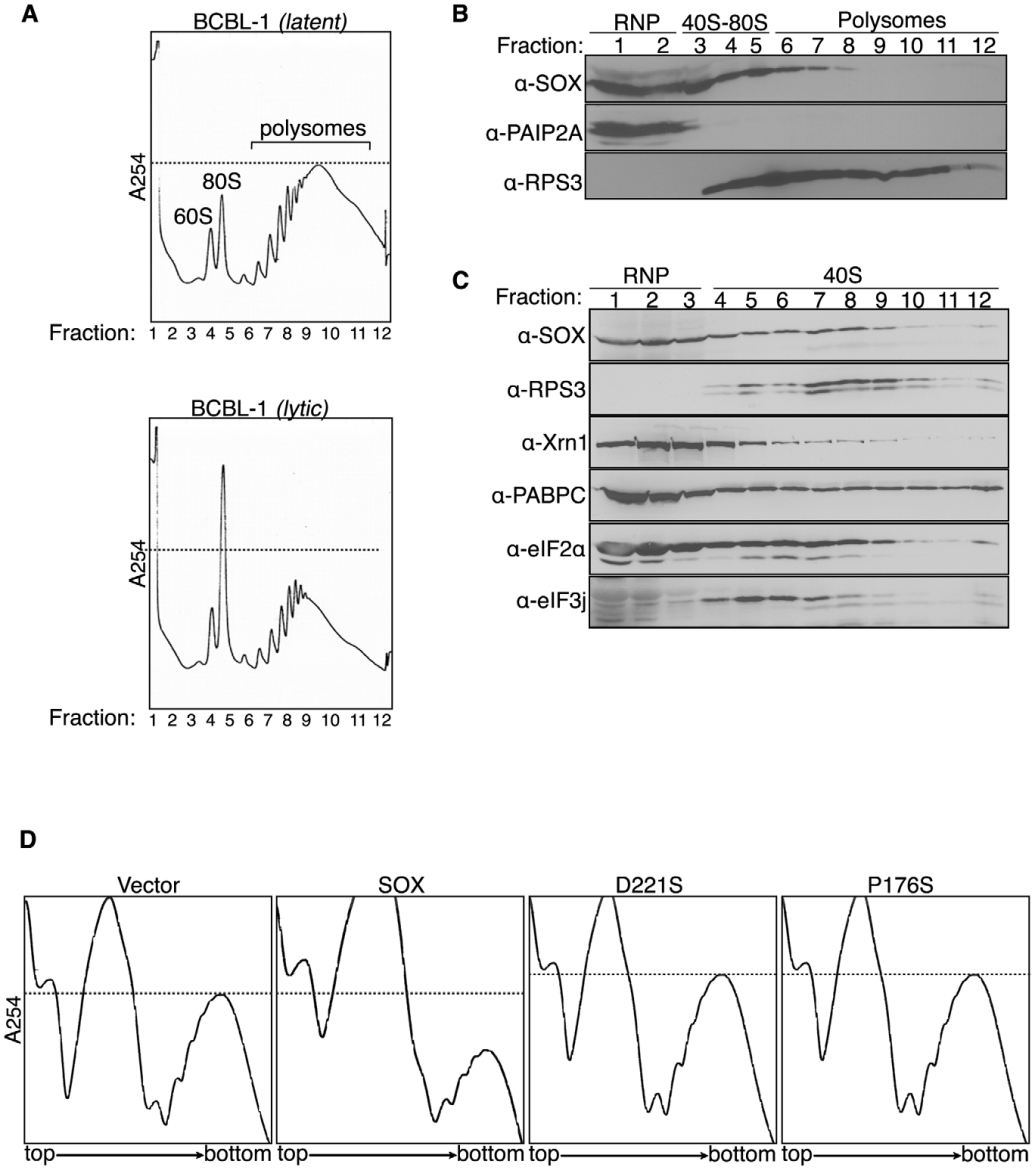
SOX depletes polyribosomes and cosediments with 40S subunits. (A) TREx BCBL-1-RTA (BCBL-1) cells were mock treated (*latent*) or induced (*lytic*) with 1 µg/ml doxycycline, 500 ng/ml ionomycin, and 20 ng/ml 2-*O*-tetradecanoylphorbol-13-acetate for 24 h to stimulate KSHV replication, then subjected to sucrose gradient fractionation using a 15–60% sucrose gradient in order to monitor the abundance of translating polysomes. (B) Fractions collected from the induced BCBL-1 gradients shown in panel A were Western blotted with the indicated antibodies. (C) Lysate from induced BCBL-1 cells was fractionated using a 5–20% sucrose gradient and analyzed by Western blot with indicated antibodies. (D) 293T cells were transfected with the indicated construct and subjected to sucrose gradient fractionation to obtain polysome profiles. Dashed lines indicate polysome levels of either latent cells (*A*) or vector expressing cells (*D*).

To examine the effects of host shutoff on translating mRNAs, we performed polysome profiling of a B cell line (BCBL-1) derived from a patient with primary effusion lymphoma, which harbors KSHV in a latent state. We used a line of BCBL-1 cells bearing an inducible version of the KSHV major lytic transactivator RTA (TREx BCBL-1-RTA) [45] to allow efficient lytic reactivation following RTA induction. Upon chemical stimulation of lytic KSHV replication in these cells (when SOX is expressed from the viral genome), we observed a significant decrease in the polysome population and a corresponding increase in 80S monosomes, consistent with degradation of actively translating mRNAs (Figure 5A). It should be noted that the level of polysome depletion during viral infection is likely underestimated, as ∼20% of induced cells generally fail to enter the lytic cycle. The depletion of polyribosomes was not due to chemical treatment alone, because treatment of the KSHV negative BJAB B cell line did not result in a similar decrease in polysomes (data not shown). We next looked for the presence of SOX in gradient fractions from the corresponding polysome profiles of lytically reactivated BCBL-1 cells. As controls, we also blotted for PAIP2A, a protein not found in polysomes [46], as well as the ribosomal protein RPS3 (Figure 5B). SOX appeared to sediment primarily with the ribonucleoprotein (RNP), 40S, and monosome fractions, and exhibited partial overlap with RPS3 (Figure 5B). Puromycin treatment disrupted polysomes but failed to alter the SOX sedimentation profile, arguing against a specific association with the 80S and polysome fractions (data not shown). To more accurately determine its sedimentation profile, we increased the resolution of the lighter molecular weight complexes using lower density sucrose gradients. These experiments revealed that SOX indeed sediments in both the RNP and 40S fractions, similar to Xrn1 (Figure 5C). Its sedimentation profile also overlapped with the eIF3j and eIF2α components of the 40S pre-initiation complex, which is recruited to the 5′ cap prior to ribosomal scanning (Figure 5C). This is in contrast to the sedimentation of PABPC, which remains bound to the mRNA throughout the polysome fractions as well (Figure 5C; data not shown). Similar data were obtained upon transient expression of SOX in 293T cells (Figure S5A). We also tested the sedimentation profile of the SOX D221S catalytic mutant, and found it to mimic that of wild-type SOX (Figure S5B), indicating that the catalytic site of the protein is unlikely to be involved in its recruitment to target RNAs.

Given that SOX is the dominant effector of host shutoff during KSHV infection, we hypothesized that polysome depletion in BCBL-1 cells was a consequence of SOX activity. We therefore tested the effects of SOX on the endogenous translating mRNA pool through polysome profiling of 293T cells expressing either wild-type SOX, the SOX catalytic mutant D221S, or the single function SOX P176S mutant lacking host shutoff but not DNase activity. Indeed, expression of wild-type SOX alone caused a significant depletion of polysomes relative to vector-transfected cells, whereas neither of the host shutoff defective SOX mutants had this effect (Figure 5D). These results additionally confirm that the catalytic activity of SOX is required to initiate widespread turnover of endogenous host messages.

### SOX specifically targets mRNAs at an early stage of translation

We had previously observed that a translationally incompetent version of the GFP mRNA, which terminates by ribozyme cleavage and lacks a poly(A) tail (GFP-HR) (Figure S6A), escaped SOX-mediated turnover [25]. Although this mRNA is inefficiently exported, subcellular fractionation experiments confirmed that even the exported cytoplasmic population of GFP-HR was not subject to SOX-induced turnover (Figure S6B). Thus, the failure of SOX to degrade this mRNA in the cytoplasm could be due to its translational incompetence.

**Figure 6 ppat-1002339-g006:**
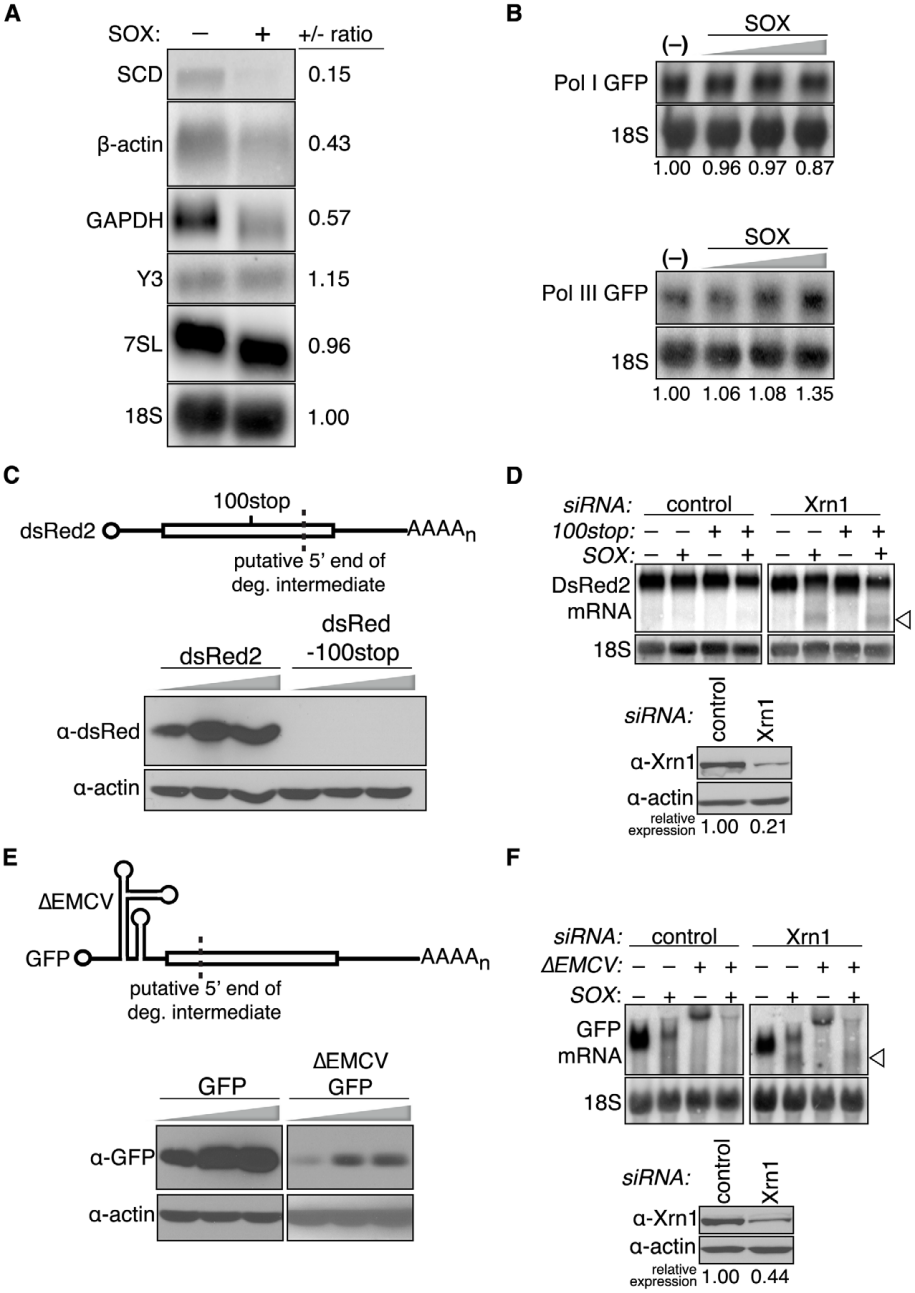
SOX targets mRNA at an early stage of translation. (A) 293T cells were transfected with control or Xrn1 shRNA-expressing constructs, and subsequently with either GFP or GFP-SOX. Cells were then sorted for GFP fluorescence to generate a pure population of SOX-expressing and control cells prior to RNA extraction. RNA was Northern blotted with the indicated probes against endogenous Pol I (18S), Pol II (SCD, β-actin, GAPDH), and Pol III (Y3, 7SL) RNAs. (B) 293T cells were transfected with either Pol I-driven GFP or Pol III-driven GFP reporters with or without increasing amounts of SOX (200–600 ng), then total RNA was Northern blotted using GFP or 18S probes. (C) 293T cells were transfected with increasing amounts (100–300 ng) of dsRed2 or a dsRed2–100stop construct containing a premature termination codon upstream of the predicted cleavage site (dashed line), then Western blotted for dsRed protein. (D) 293T cells were transfected with control or Xrn1 shRNAs and subsequently with either the dsRed2 or dsRed2-100stop (100stop) reporter in the presence or absence of SOX. RNA was Northern blotted with 3′ end dsRed2 or 18S probes. The arrowhead indicates the position of the SOX-induced cleavage product. (E) 293T cells were transfected with increasing amounts (100–300 ng) of GFP or a ΔEMCV-GFP construct containing the ΔEMCV IRES in the 5′ UTR, then Western blotted for GFP protein. (F) 293T cells were transfected with control or Xrn1 shRNAs and then with either the GFP or ΔEMCV-GFP reporter in the presence or absence of SOX. RNA was Northern blotted with 3′ GFP or 18S probes. The arrowhead indicates the position of the SOX-induced cleavage product. The Western blot shows level of Xrn1 depletion, and the actin loading control.

To further explore a role for translation in SOX-induced mRNA turnover, we examined SOX turnover of RNAs transcribed by Pol I and Pol III. Pol I and Pol III transcription does not result in the addition of the cap and poly(A) tail, mRNA modifications critical to translation initiation specifically associated with Pol II transcription. Using a pure population of cells expressing GFP-SOX or GFP alone, we found that neither the endogenous Pol III-generated Y3 and 7SL cytoplasmic non-coding RNAs, nor the 18S rRNA transcribed by Pol I undergo turnover in the presence of SOX (Figure 6A). In contrast, we observed significant depletion of endogenous mRNAs transcribed by Pol II, including GAPDH, β-actin, and stearoyl-CoA desaturase (SCD) in SOX-expressing cells (Figure 6A). In principle, the inability of SOX to degrade the non-Pol II transcripts could be due to the absence of an ORF, or the presence of RNP complexes occluding SOX cleavage sites. To exclude these possibilities, we expressed the GFP reporter under the control of Pol I or Pol III promoters and found that in both cases, the GFP RNA was not targeted by SOX (Figure 6B). We confirmed using subcellular fractionation experiments that the inability of SOX to promote degradation of these RNAs was not due to a failure of the RNAs to be exported (Figure S6E and S6F). Collectively, these data indicate that RNAs must be translationally competent to be targeted by SOX.

To determine whether mRNA degradation by SOX required ribosomal passage over or near the putative initial cleavage site, we designed a reporter dsRed2 mRNA harboring a termination codon upstream of the putative SOX cleavage site (dsRed2-100stop). This prevented production of full-length dsRed2 protein (Figure 6C). Interestingly, in cells depleted of Xrn1, dsRed2-100stop was cleaved similarly to wild-type dsRed2 upon SOX expression (Figure 6D), indicating that ribosomal passage over the cleavage site is not necessary for its recognition.

To determine whether 60S joining is required for cleavage, we made use of a modified version of the encephalomyocarditis virus internal ribosomal entry site (IRES), termed ΔEMCV, which cannot promote cap-independent translation but is highly structured and decreases translation initiation *in vitro* [47]. Indeed, insertion of ΔEMCV into the GFP 5′ UTR 30 nt from the cap significantly reduced GFP protein accumulation (Figure 6E). However, in Xrn1-depleted cells, SOX still induced cleavage of this mRNA with similar efficiency as wild-type GFP, and at the same site (Figure 6F). In agreement with the ΔEMCV-GFP data, we observed that SOX could also promote turnover of a GFP mRNA with a 60 nt adenylate tract inserted in the 5′ UTR (5′A60-GFP) (Figure S6D), which similarly reduced GFP protein production (Figure S6C). A 50–70 nt adenylate-rich tract in the 5′ UTR of PABPC has likewise been shown to repress translation of its message in an autoregulatory manner as a consequence of PABPC protein binding to this region [48]. Consistent with the sedimentation profile of SOX, these data suggest that in SOX-expressing cells, mRNAs are targeted for cleavage at an early step during translation, perhaps prior to AUG recognition.

**Figure 7:**
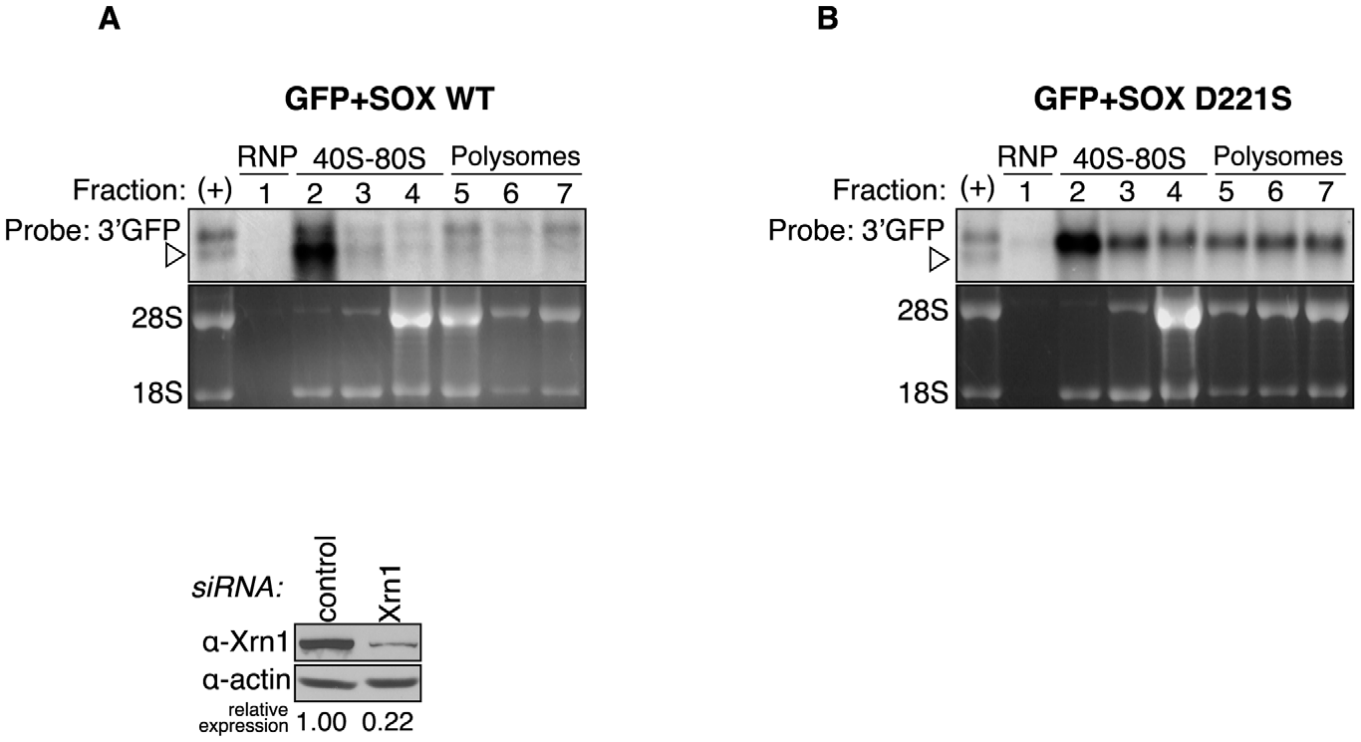
The degradation intermediate sediments predominantly with the 40S fractions. 293T cells were transfected with Xrn1 shRNA, followed by expression of GFP and either wild-type (WT) SOX (A) or the catalytically dead SOX D221S (B). They were then fractionated over sucrose gradients and RNA from each fraction was Northern blotted with a 3′ GFP probe. Ribosomal RNA was visualized by ethidium bromide staining. In both panels the (+) lane shows the migration of full-length GFP and the degradation intermediate in unfractionated RNA from cells expressing wild-type SOX. It was used as a reference to identify the different RNA species in the fractionated RNA. Arrowheads point to degradation intermediates.

To monitor directly where cleavage occurs, we performed sucrose density gradient centrifugation on Xrn1-depleted cells expressing SOX and the GFP reporter. In agreement with our above findings, the cleaved intermediate preferentially accumulates in the 40S fraction (Figure 7A). The degradation intermediate is absent in all fractions from cells expressing the SOX catalytic mutant D221S (Figure 7B). These results lead us to favor a model in which mRNAs are recognized by SOX during formation of the 40S preinitiation complex, at which point they undergo SOX-induced endonucleolytic cleavage and subsequent destruction by Xrn1.

## Discussion

### KSHV SOX and hXrn1 coordinate mRNA decay

This study shows that the global destruction of cellular mRNA during KSHV-induced host shutoff is enacted through the coordinated activities of both the viral SOX protein and cellular Xrn1. Our findings suggest that, in cells, SOX directs endonucleolytic cleavage of mRNAs at an early stage during translation, and subsequently employs host nucleases such as Xrn1 to execute degradation of the mRNA body. Thus, the virus is able to make use of the available host mRNA turnover machinery, yet bypass the rate limiting events that normally precede activation of Xrn1. Collectively, these data and our previous studies reconcile the seemingly disparate observations that the gammaherpesvirus SOX homologs promote potent host shutoff, yet exhibit relatively weak *in vitro* RNase activity. We hypothesize that while SOX can catalyze RNA cleavage, it does so in a site-specific manner in cells and requires one or more cellular factors to recruit and/or position it on the mRNA substrate, as well as host enzymes to complete the degradation process.

### Evidence for SOX as an endonuclease

Endonucleolytic cleavages enable rapid mRNA turnover, because they generate unprotected RNA ends while bypassing the requirement for deadenylation and decapping. Cellular endonucleases are therefore generally subject to tight regulation. In eukaryotic cells, endonuclease-mediated decay is associated with mRNA quality control pathways, such as NMD, which targets messages containing premature stop codons [36], [37], and No-Go decay (NGD), which destroys mRNAs that experience stalls in translation [49]. In addition, recent studies have described previously unappreciated contributions of eukaryotic endonucleases to other basic processes such as miRNA-mediated mRNA decay [50], ER stress responses [51], [52], and exosome-mediated decay [53], [54], [55]. A few endonucleases that regulate decay of specific sets of otherwise normal mRNAs have also been described [10].

Several observations suggest that a primary role of SOX during host shutoff is to mediate endonucleolytic cleavage of target mRNAs, thereby enabling direct access by Xrn1. A key finding is that Xrn1-depleted cells undergoing host shutoff accumulate defined size degradation intermediates, similar to those derived from transcripts undergoing NMD or NGD under conditions of limiting Xrn1 [36], [37], [42], [49], [56]. Additionally, the observation that deadenylation and Dcp1/2-mediated decapping are not required to generate the degradation intermediate suggest that the 5′ and 3′ ends of the mRNA remain intact at the time of cleavage. We predict that SOX induces a sequence-specific rather than a position-dependent cut, as duplicating a 201 nt region surrounding the cut site yielded a second degradation intermediate, and addition of sequences upstream of the cleavage site did not alter the cleavage efficiency or location. We identified a conserved stretch of five nucleotides just upstream of the cut site in three different reporters. This consensus site was necessary, but not sufficient to elicit cleavage, suggesting that it represents a portion of a larger element required for SOX targeting. We hypothesize that the full element is a complex sequence or structure, perhaps degenerate, enabling it to be widespread among cellular mRNAs.

It is likely that additional enzymes besides Xrn1 participate in clearance of the fragments created by SOX, particularly in removal of the 5′ degradation intermediate. However, we have not observed a role for the canonical 3′-5′ exosome exonuclease complex. One possibility is that another host 3′-5′exonuclease instead mediates degradation of the upstream fragment, or plays a compensatory role upon exosome depletion. This might explain the limited protection of the 5′ fragment afforded by exosome knockdown in studies of another eukaryotic endonuclease [36]. Alternatively, more robust depletion of the exosome might be required to inhibit its activity in the context of cytoplasmic mRNA degradation.

SOX bears significant structural similarity to the PD-(D/E)XK type II restriction endonuclease superfamily [41], which includes several proteins that have been experimentally demonstrated to have endonuclease activity on RNA [57], [58]. The catalytic residues of SOX are essential for mRNA turnover in cells, further supporting a direct role for SOX in the primary cleavage event. Interestingly, *in vitro* RNase activity has recently been described for SOX and its EBV homolog BGLF5, although these studies concluded that it functioned as an exonuclease [28], [30]. However, RNases can harbor both endonuclease and exonuclease activity in the same active site [59], [60]. In addition, our data indicate that the endonucleolytic activity of SOX occurs in a site-specific manner, whereas the published assays used generic RNAs.

### Mechanism of mRNA targeting

Our results support the involvement of an early stage of translation in the targeting of mRNA by SOX. This would allow the virus to selectively eliminate competing host mRNAs, while sparing regulatory RNAs that may be necessary for its own gene expression. Eukaryotic translation initiates with the recruitment of the 40S ribosomal subunit to the cap via interactions with translation initiation factors, followed by scanning to the AUG codon and recruitment of the 60S subunit. Two observations indicate that recognition and turnover in SOX-expressing cells are initiated prior to 60S joining. Specifically, insertion in the 5′ UTR of elements that presumably inhibit or prematurely terminate 40S scanning, and thus significantly reduce protein production, does not affect SOX activity. Moreover, the degradation intermediate accumulates preferentially in the 40S fraction. Our finding that SOX cosediments with the 40S initiation complex suggests that an association with translation initiation machinery directs it to mRNAs. Supporting this prediction is the failure of SOX to target translationally incompetent reporter and endogenous RNAs transcribed by RNA polymerase I or III.

We hypothesize that SOX targeting requires recognition of the mRNA 5′ end, likely via associated translation initiation factors, and that this recruitment somehow allows SOX access to the cleavage site(s) within the mRNA. Interestingly, our data show that cleavage can occur hundreds of nucleotides away from the site of translation initiation. One interesting parallel to these seemingly discrepant observations is that of the *E. coli* endonuclease RNase E, which can cleave anywhere along the length of the RNA (preferably within AU-rich sequences), but requires a monophosphate at the mRNA 5′ end. Thus, similar to our proposed model for SOX activity, RNase E can simultaneously recognize two non-adjacent regions of the primary RNA sequence [61]. These observations can be reconciled by the fact that RNAs adopt secondary and tertiary structures within a cell that could presumably enable such sequences to be juxtaposed. Alternatively, this distance could be bridged if there was an additional interaction of SOX with a host protein co-factor bound to the cleavage element.

### Implications for virus-induced host shutoff

While this is the first example of a viral protein working in concert with host RNA turnover components to broadly target cellular messages, it is likely that several other viruses use similar mechanisms to enact host shutoff. Alphaherpesviruses and SARS coronavirus also encode host shutoff factors (vhs and nsp1, respectively) that promote mRNA degradation, but bear no homology to SOX [16], [17]. *In vitro* data indicate that they similarly induce endonucleolytic cleavage of RNAs, and it has been proposed that degradation of the mRNA body in cells may be assisted by host exonucleases, although this has yet to be shown [62], [63], [64], [65], [66]. In addition, all three viral proteins appear to use components of the translation machinery as a means to access mRNA [62], [65], [67], [68], [69]. Such parallels suggest that these viruses have adopted remarkably similar strategies to execute mRNA decay, resembling those of host pathways like NMD. In addition to the enhanced rate of decay afforded by an endonucleolytic mechanism, the advantage for the virus may be that this strategy of host shutoff generates intermediates that look indistinguishable from products of quality control pathways. Such intermediates would then be readily degraded by the core degradation machinery and might not be recognized as aberrant, perhaps avoiding activation of stress or innate immune responses. Thus, understanding how viral endonucleases interface with host pathways may provide insight into how manipulation of these pathways contributes to infectious disease, as well as into events that regulate cellular RNA decay.

## Materials and Methods

### Cell Extracts, Western blots, and Northern Blots

Protein lysates were either prepared in RIPA buffer [50 mM Tris-HCl (pH8.0), 150 mM NaCl, 1% (v/v) Nonidet P-40, 0.5% (w/v) sodium deoxycholate, 0.1% (w/v) sodium dodecyl sulfate (SDS)], or fractionated using the NE-PER kit (ThermoScientific). Western blots were performed with either mouse monoclonal anti-GFP (1∶2000, BD Biosciences), mouse monoclonal anti-dsRed (1∶500, Clontech), mouse monoclonal anti-HA (1∶2000, Invitrogen), mouse monoclonal anti-RPS3 (1∶1000, Abcam), mouse monoclonal anti-Flag (1∶1000, Sigma), rabbit polyclonal anti-PAIP2A (1:2000, kindly provided by N. Sonenberg [70]), rabbit polyclonal anti-hXrn1 (1∶5000, kindly provided by J. Lykke-Andersen), rabbit polyclonal anti-hDcp1a (1∶5000, kindly provided by J. Lykke-Andersen), rabbit polyclonal anti-hDcp2 (1∶400, kindly provided by M. Kiledjan), rabbit polyclonal anti-hRrp41 (1∶1000, see below), rabbit polyclonal anti-SOX J5803 (1∶5000, [33]), mouse monoclonal hnRNPC1/C2 (1∶2000, Abcam), mouse monoclonal anti-Hsp90 (1∶3000, Stressgen Bioreagents), mouse monoclonal anti-tubulin (1∶3000, Sigma Aldrich), goat polyclonal anti-actin (1∶5000, Santa Cruz Biotechnology), mouse monoclonal 10e10 anti-PABPC (1∶2000, Santa Cruz Biotechnology), rabbit polyclonal anti-eIF2α (1∶1000, Cell Signaling) or rabbit polyclonal anti-eIF3j (1∶1000, Cell Signaling). Rabbit polyclonal antibodies were raised against recombinant maltose binding protein (MPB)-fused hRrp41 purified from *E. coli*.

Total cellular RNA was isolated for Northern blotting using RNA-Bee (Tel-Test). Where indicated, the NE-PER kit (ThermoScientific) was used for cellular fractionation prior to RNA extraction. Northern blots were probed with ^32^P-labeled DNA probes made using either RediPrime II (GE Healthcare) or Decaprime II (Ambion) or, for β-globin, an SP6 transcribed ^32^P-labeled riboprobe. RNase H assays were carried out as previously described [25]. Results in each figure are a representative of at least 3 independent replicates of each experiment. Image J (http://rsbweb.nih.gov/ij/) was used for quantification of Northern and Western blots.

### Polysome profiling

Profiles were obtained from uninduced or lytically reactivated TREx BCBL-1-RTA cells, or from 293T cells transfected with the indicated plasmids. Polysome profiles were carried out as described in Jackson and Larkins [71], except that cells were treated with 100 µg/ml CHX for 30 minutes prior to harvesting. BCBL-1 and 293T extracts were pelleted through 60% sucrose before layering or were directly layered on a 15–60% sucrose gradient containing 100 µg/ml CHX. Additional details, including the procedure for resolution of RNP/40S fractions are described in supplemental procedures (Text S1).

Additional experimental procedures, primer and siRNA/shRNA sequences used in the study are detailed in Supplemental Materials and Methods (Text S1).

## Supporting Information

Figure S1 **Further evidence for a role for Xrn1 in SOX-mediated mRNA turnover using additional reporters.** (A–B) 293T cells were treated with control or Xrn1 siRNAs and transfected with DsRed-Express-DR (A) and β-globin (B) in the presence or absence of SOX. mRNA was subjected to Northern blotting using 3′ end-directed probes. DsRed-Express-DR bears different 5′ and 3′ UTRs from DsRed2, and has minor changes in the coding region. 18S was used as a loading control. Arrowheads denote degradation intermediates. The corresponding Western blot showing the level of Xrn1 protein depletion is shown in Figure 1B. (C–D) A flaviviral Xrn1-blocking element (SLII) was inserted at different positions within the GFP mRNA, as indicated in the diagram (C). 293T cells were transfected with the reporters in the presence or absence of SOX. Total RNA from the indicated samples was Northern blotted using a probe directed against the 3′ UTR of the GFP reporter or 18S (D). Arrowheads denote protected fragments. (E) The SLII element was inserted in the 3′ UTR of β-globin. 293T cells were transfected with the regular β-globin reporter or with the 3′UTR-SLII bearing reporter in the presence or absence of SOX. Total RNA from the indicated samples was Northern blotted using a probe directed against the 3′ UTR of the β-globin reporter or 18S. Arrowhead denotes the protected fragment.
(EPS)

Figure S2 **mRNA in SOX-expressing cells is not stabilized by depletion of proteins involved in decapping or 3′**–**5′ degradation.** (A) The GFP-2xSLII construct, a modified GFP reporter containing two copies of the flaviviral Xrn1-blocking element SLII within the coding region was used to test the effect on decapping of the Dcp2 E148Q mutant. As shown in Figure 2A, the insertion of two SLII elements results in accumulation of protected RNA fragments even in the absence of SOX, presumably as a consequence of basal deadenylation- and decapping-dependent turnover by Xrn1. Thus, if the Dcp2 dominant negative (DN) mutant inhibits decapping of this mRNA, the appearance of the Xrn1-protected fragment should be diminished. (B) 293T cells were transfected with GFP-2xSLII and with increasing amounts of the Flag-tagged Dcp2 E148Q mutant (Flag-Dcp2 DN, 0–800 ng). Total RNA was Northern blotted with a 3′ UTR GFP or an 18S probe. The image contrast and brightness were enhanced (*middle panel*) to highlight the shorter RNA species (arrowheads), as in the absence of SOX only a minor proportion of the full-length mRNA undergoes decay during the course of the experiment. Western blotting confirmed expression of the Dcp2 DN protein (*bottom panel)*. The appearance of the protected fragments is reduced in the presence of the Dcp2 E148Q, indicating that Dcp2 E148Q is acting as a dominant negative mutant, in that it prevents the formation of decapped, Xrn1-accessible substrates during basal mRNA decay. (C) 293T cells were treated with control siRNAs or siRNAs against the decapping complex proteins Dcp1A and Dcp2, or the exonuclease Xrn1. They were then transfected with GFP in the presence of absence of SOX, and with the Dcp2 dominant negative E148Q (Dcp2 DN) construct where indicated. Total RNA was Northern blotted using a 3′ UTR probe against GFP or 18S. Western blots demonstrate level of protein depletion after siRNA treatment and confirm Dcp2 DN overexpression. Actin was used as a loading control. Grey lines indicate where intervening lanes were cropped out of the image. (D) 293T cells were treated with control or hRrp41 siRNAs, then transfected with GFP in the presence or absence of SOX. Total RNA was Northern blotted using a 3′ UTR probe against GFP or 18S (*left panels*). Western blots demonstrate level of depletion of hRrp41 upon siRNA treatment, using actin as a loading control (*right panels)*. (E) 293T cells were treated with control siRNAs, or siRNAs against Xrn1 or hRrp41, then transfected with DsRed2 in the presence or absence of SOX. Total RNA was Northern blotted using a 5′ or 3′ UTR probe against DsRed2 or 18S (*left panels*). The arrowhead denotes degradation intermediates. The diagram shows the expected size of the 5′ and 3′ degradation intermediate generated by cleavage of the mRNA in the presence of SOX (*top*). Western blots demonstrate level of depletion of Xrn1 and hRrp41 upon siRNA treatment, using actin as a loading control (*right panels)*.
(EPS)

Figure S3 **5′ RACE analysis of the degradation intermediate reveals a predominant cleavage site preceded by a conserved 5-mer.** The enriched band from the 5′ RACE PCR (see Figure 3D) was cloned and sequenced for each of the indicated reporters. Numbers above the sequence indicate nucleotide position with respect to translational start size. Numbers below the sequence indicate the number of clones sequenced that ended at the marked nucleotide. “n” indicates the number of clones sequenced for each construct. The conserved 5-mer preceding the cleavage sites is underlined.
(EPS)

Figure S4 **The predicted catalytic and DNA binding residues of SOX are required for DNase activity.** DNase assays were conducted to monitor the ability of in vitro translated ^35^S-labeled SOX proteins to degrade linearized plasmid DNA. Mutation of any of the catalytic residues (A) or of the putative DNA binding residues (B) abolishes DNase activity (*top panels*). Autoradiography was used to confirm that the SOX mutant proteins were expressed at least to the level of wild-type SOX (*bottom panels*) (C) The position of the mutated residues were modeled within the SOX structure. The PDB coordinates (3FHD) from the structure of SOX as determined by Dahlroth et al. [41] were entered in MacPymol to generate a pictorial representation of the structure. Putative catalytic residues are shown in red (coordinated around a Mg^2+^ ion) and putative DNA-binding residues are in blue. All marked residues were largely required for DNase activity as shown above. All the catalytic residues (E184, D221, E244, K246) were required for host shutoff (Figure 4A). In contrast, of the DNA binding residues, some (R139, S144, Q376) were required for host shutoff, while others (W135, S146, S219) were dispensable (Figure 4B).
(TIF)

Figure S5 **Transiently expressed SOX in 293T cells cosediments with translation initiation factors.** Lysates from 293T cells transfected with wild-type SOX (A) or the catalytic mutant SOX D221S (B) were analyzed by sucrose gradient fractionation through a 5–20% sucrose gradient. SOX proteins were expressed to near-physiological levels using the 3′ Moloney murine leukemia virus long terminal repeat (LTR) promoter in the pBMN vector. Fractions were analyzed by Western blot with indicated antibodies.
(TIF)

Figure S6 **Evaluation of SOX-induced turnover of translation-defective mRNA constructs.** (A) 293T cells were transfected with either normal GFP or a GFP reporter terminating in a hammerhead ribozyme, generating a transcript that is not polyadenylated (GFP-HR; shown in diagram, arrow points at site of ribozyme cleavage). GFP protein expression was assessed by Western blot analysis. Actin was used as a loading control. (B) 293T cells were co-transfected with the normal GFP reporter and GFP-HR with or without SOX. Cells were divided into nuclear and cytoplasmic fractions from which total RNA and protein were extracted. RNA was Northern blotted with a probe annealing to the first 300 bp of the GFP coding region or an 18S probe (upper panels), and protein lysates were Western blotted with α-hnRNP-C1 and α-Hsp90 antibodies to assess the purity of nuclear/cytoplasmic fractions (lower panels). (C) 293T cells were transfected with increasing amounts (100–300 ng) of GFP or a 5′A60-GFP construct containing a stretch of adenosines in the 5′ UTR (depicted in diagram). Western blot demonstrates a significant reduction in translation of 5′A60-GFP. (D) HEK 293T cells were transfected with the 5′A60-GFP reporter with or without increasing amounts of SOX (100–300 ng). Total RNA was Northern blotted with GFP probes or 18S probes. (E–F) 293T cells were transfected with either Pol I-driven GFP (E) or Pol III-driven GFP (F) reporter constructs in the absence or presence of SOX. Samples were fractionated into nuclear and cytoplasmic fractions and processed for RNA and protein as described in (B). RNA was Northern blotted with GFP and 18S probes, and protein lysates were Western blotted with the indicated antibodies.
(TIF)

Text S1 **Supplemental Material and Methods.** Includes additional details on experimental procedures and a list of primers, siRNA and shRNAs used in the study.
(DOC)
