# Supplementary figures and images for: Coordinated Destruction of Cellular Messages in Translation Complexes by the Gammaherpesvirus Host Shutoff Factor and the Mammalian Exonuclease Xrn1
Source: PLoS Pathog. 2011 Oct 27;7(10):e1002339. doi: 10.1371/journal.ppat.1002339 (PMC3203186; doi:10.1371/journal.ppat.1002339)

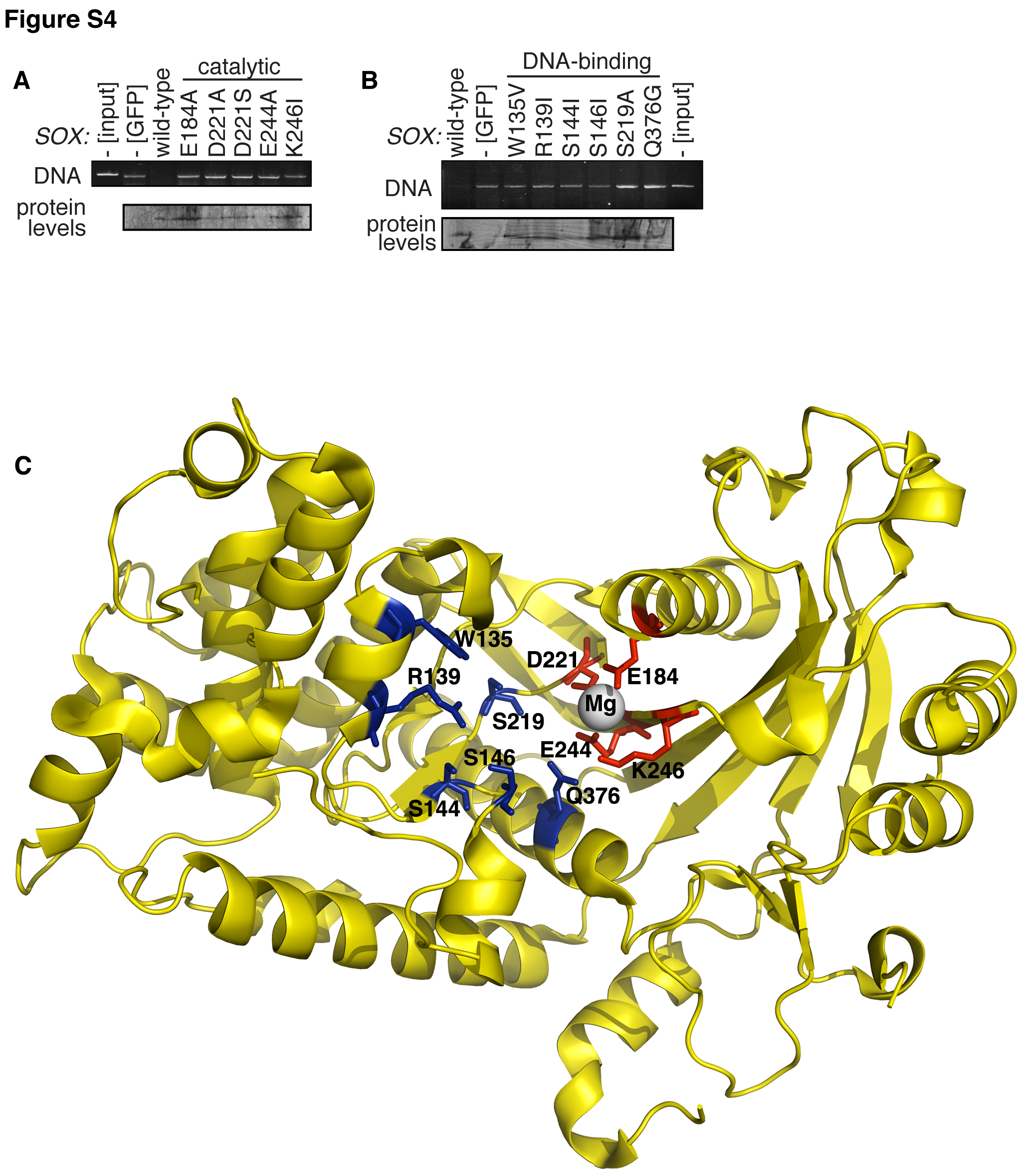

Supplement: Figure S4 — The predicted catalytic and DNA binding residues of SOX are required for DNase activity. DNase assays were conducted to monitor the ability of in vitro translated 35S-labeled SOX proteins to degrade linearized plasmid DNA. Mutation of any of the catalytic residues (A) or of the putative DNA binding residues (B) abolishes DNase activity (top panels). Autoradiography was used to confirm that the SOX mutant proteins were expressed at least to the level of wild-type SOX (bottom panels) (C) The position of the mutated residues were modeled within the SOX structure. The PDB coordinates (3FHD) from the structure of SOX as determined by Dahlroth et al. [41] were entered in MacPymol to generate a pictorial representation of the structure. Putative catalytic residues are shown in red (coordinated around a Mg2+ ion) and putative DNA-binding residues are in blue. All marked residues were largely required for DNase activity as shown above. All the catalytic residues (E184, D221, E244, K246) were required for host shutoff (Figure 4A). In contrast, of the DNA binding residues, some (R139, S144, Q376) were required for host shutoff, while others (W135, S146, S219) were dispensable (Figure 4B). (TIF) [file ppat.1002339.s004.tif]

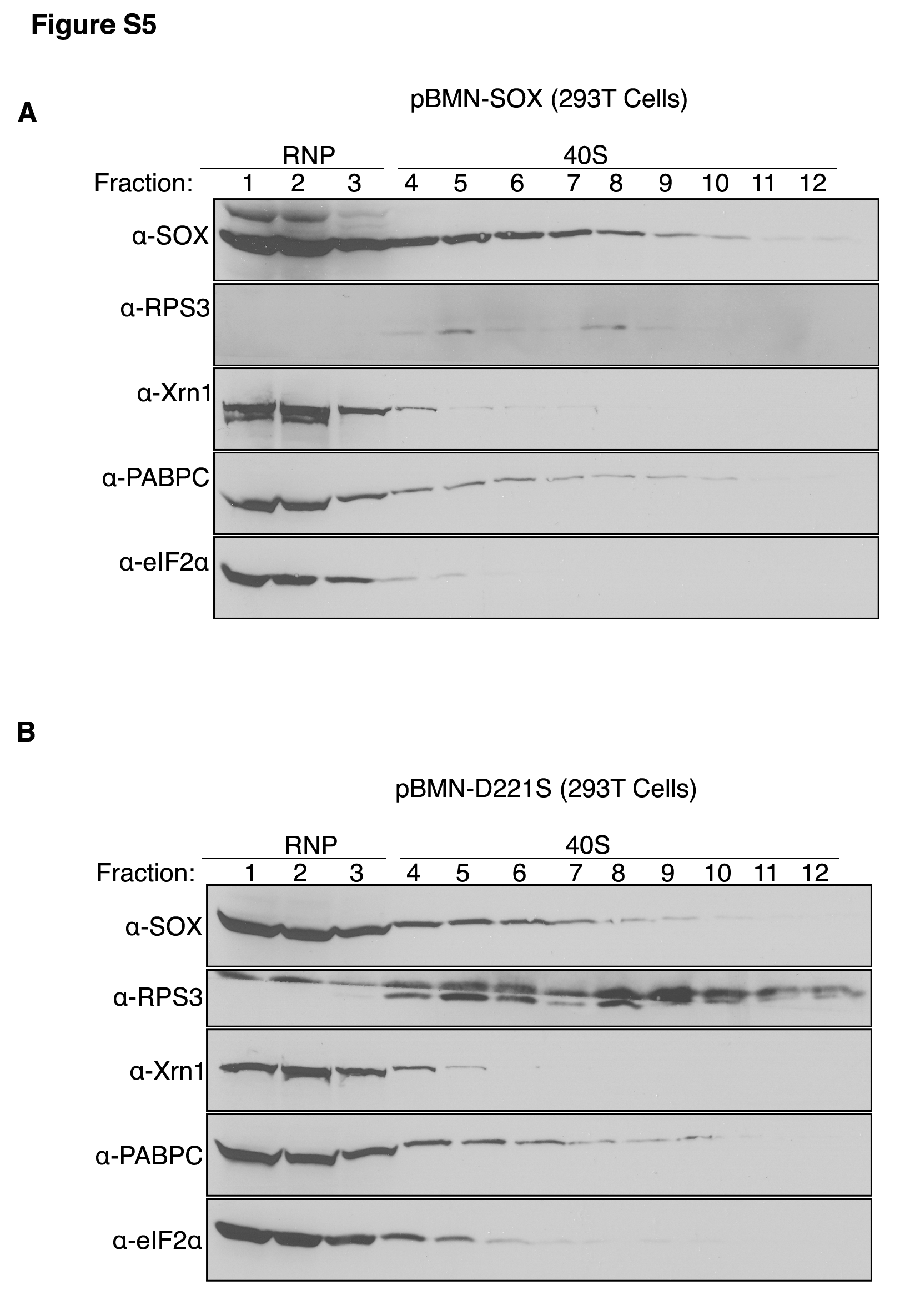

Supplement: Figure S5 — Transiently expressed SOX in 293T cells cosediments with translation initiation factors. Lysates from 293T cells transfected with wild-type SOX (A) or the catalytic mutant SOX D221S (B) were analyzed by sucrose gradient fractionation through a 5–20% sucrose gradient. SOX proteins were expressed to near-physiological levels using the 3′ Moloney murine leukemia virus long terminal repeat (LTR) promoter in the pBMN vector. Fractions were analyzed by Western blot with indicated antibodies. (TIF) [file ppat.1002339.s005.tif]

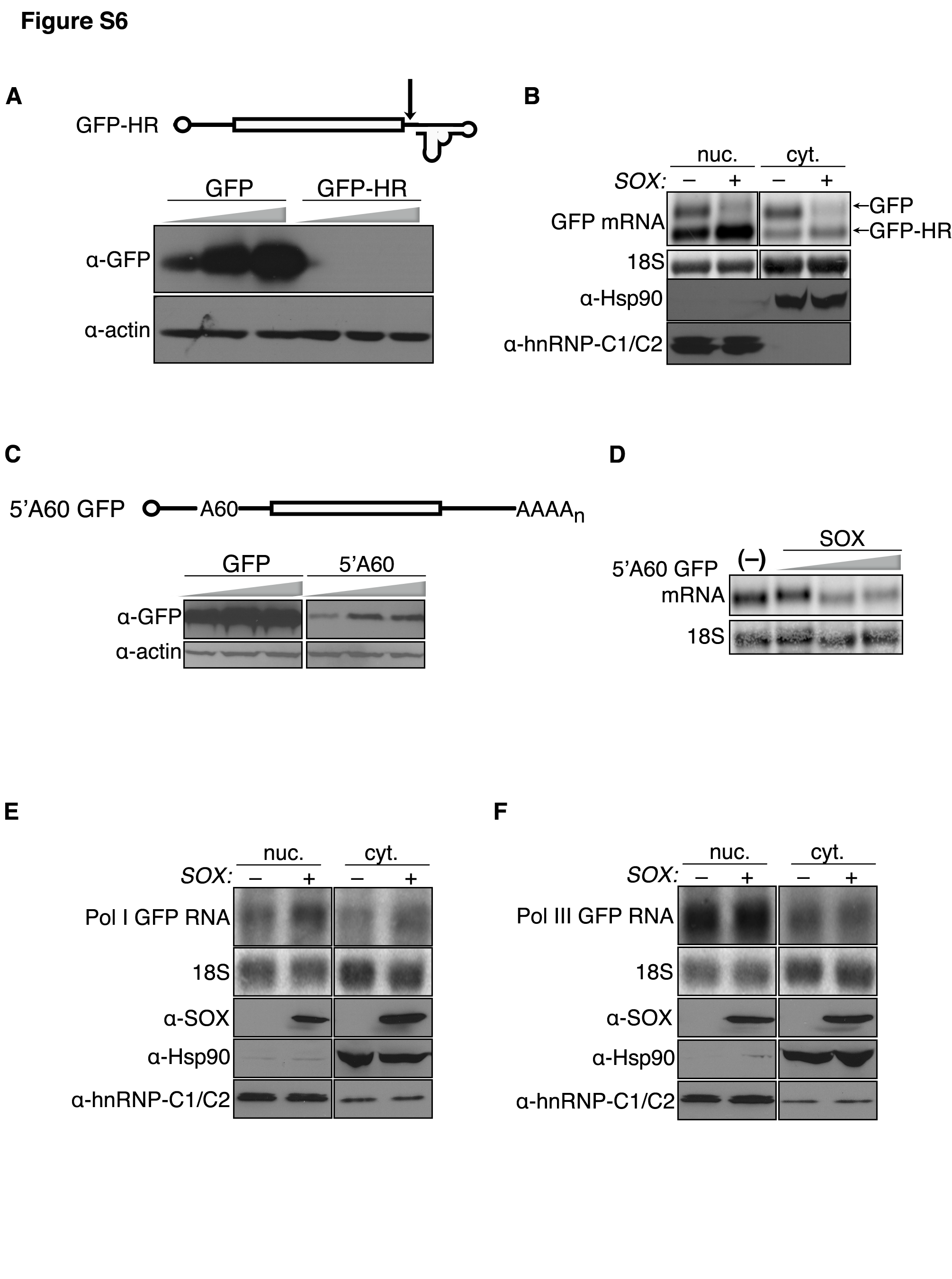

Supplement: Figure S6 — Evaluation of SOX-induced turnover of translation-defective mRNA constructs. (A) 293T cells were transfected with either normal GFP or a GFP reporter terminating in a hammerhead ribozyme, generating a transcript that is not polyadenylated (GFP-HR; shown in diagram, arrow points at site of ribozyme cleavage). GFP protein expression was assessed by Western blot analysis. Actin was used as a loading control. (B) 293T cells were co-transfected with the normal GFP reporter and GFP-HR with or without SOX. Cells were divided into nuclear and cytoplasmic fractions from which total RNA and protein were extracted. RNA was Northern blotted with a probe annealing to the first 300 bp of the GFP coding region or an 18S probe (upper panels), and protein lysates were Western blotted with α-hnRNP-C1 and α-Hsp90 antibodies to assess the purity of nuclear/cytoplasmic fractions (lower panels). (C) 293T cells were transfected with increasing amounts (100–300 ng) of GFP or a 5′A60-GFP construct containing a stretch of adenosines in the 5′ UTR (depicted in diagram). Western blot demonstrates a significant reduction in translation of 5′A60-GFP. (D) HEK 293T cells were transfected with the 5′A60-GFP reporter with or without increasing amounts of SOX (100–300 ng). Total RNA was Northern blotted with GFP probes or 18S probes. (E–F) 293T cells were transfected with either Pol I-driven GFP (E) or Pol III-driven GFP (F) reporter constructs in the absence or presence of SOX. Samples were fractionated into nuclear and cytoplasmic fractions and processed for RNA and protein as described in (B). RNA was Northern blotted with GFP and 18S probes, and protein lysates were Western blotted with the indicated antibodies. (TIF) [file ppat.1002339.s006.tif]
