# Supplementary material for: Coordinated Destruction of Cellular Messages in Translation Complexes by the Gammaherpesvirus Host Shutoff Factor and the Mammalian Exonuclease Xrn1
Source: PLoS Pathog. 2011 Oct 27;7(10):e1002339. doi: 10.1371/journal.ppat.1002339 (PMC3203186; doi:10.1371/journal.ppat.1002339)
Supplement: Text S1 — Supplemental Material and Methods. Includes additional details on experimental procedures and a list of primers, siRNA and shRNAs used in the study. (DOC) [file ppat.1002339.s007.doc]

**Text S1: Supplemental Materials and Methods**

**Plasmid Constructs**

Plasmids pCDEF3-SOX [1], pd2eGFP-HR [2] and pCDNA3.1-GFP-SOX [3] were described previously. Plasmids pd2eGFP-N1 and pDsRed2-N1 were purchased from Clontech. To generate the pd2-SOX (used in Figure S1E) construct, the GFP coding region from pd2eGFP-N1 was excised using EcoRI and NotI and replaced with the SOX coding region from pCDEF3-SOX. The constitutively expressed β-globin reporter (pcDNA3.1-β-globin) was generated by subcloning the human β-globin gene from pcTet2-βwt [4] into the HindIII/ApaI sites of pcDNA3.1(+). Plasmid pcDNA3-FLAG-hDcp2:E148Q was kindly provided by J. Lykke-Andersen [5]. Plasmid pcDNA3.1-DsRed-Express (kindly provided by C. Sullivan) was generated by subcloning the DsRed-Express DR gene (Clontech) into the BamHI/NotI sites of pcDNA3.1. Plasmid pd2-5’A60-GFP-N1 was constructed by cloning a stretch of 60 adenosines into the 5’ UTR of GFP-N1 via the EcoRI/KpnI sites. The pd2-∆EMCV-GFP-N1 construct was made by XhoI/EcoRI digestion of the SV40 dual luciferase plasmid pR/∆E/F(C-53), kindly provided by P. Sarnow [6]. The ∆EMCV fragment was then subcloned into the 5’ UTR of GFP-N1 using XhoI/EcoRI. To generate the RNA Pol I-driven GFP construct, we subcloned the GFP open reading frame into the BsmBI sites of the pHH21 vector (kindly provided by J. Doudna) which contains the human RNA Pol I promoter and the mouse RNA Pol I terminator [7]. In order to express GFP via a Pol III promoter, the Y3 gene was first excised from the pBSU6-Y3IP construct (kindly provided by K. Collins, [8]) using PstI/BamHI. GFP was then amplified with primers containing NsiI/BglII restriction sites and subcloned into the empty vector. For the SLII constructs, one or two copies in tandem of the flaviviral Xrn1-blocking element SLII [9] from West Nile virus strain NY99 (Genbank # DQ211652.1) were cloned into pd2eGFP-N1, pDsRed2-N1 and pcDNA3.1-β-globin using either BamHI for insertion into the 5’ UTR, BsrGI for in frame insertion into the coding region (GFP only), or NotI for insertion into the 3’ UTR. A modified Quikchange Mutagenesis (Agilent) protocol was used to insert the SLII element in the DsRed2 coding region [10]. Briefly, the SLII was amplified with primers that contained sequences from the DsRed2 coding region and the PCR fragment was subsequently used to prime the Quikchange reaction. pd2eGFPrep, containing an internal repeat of the putative cleavage site within GFP, was generated by cloning in frame a copy of the 201 bp segment starting at the ATG of the GFP coding region into the downstream BsrGI site of the pd2eGFP-N1 construct. The GFP variant containing a repeat of 25 basepairs (nt 126-150 from the GFP ATG) was generated in the same way. The GFP variants carrying a 6-mer deletion (nt 133-138, GFP-∆TGAAG) or an 8-mer insertion (nt 131-138 including the TGAAG sequence, inserted right after the BsrGI site) were created using Quikchange mutagenesis. A premature termination codon at DsRed2 amino acid 100 was introduced using QuikChange to generate pDsRed2-100stop. The SOX point mutants were generated from pCDEF3-T7-SOX using QuikChange. Wild-type SOX was cloned into the EcoRI/SalI sites of pBMN-IP to generate pBMN-SOX. pBMN-D221S was generated using Quikchange.

QuikChange primer sequences are listed below in Table S1. Cloning primer sequences are listed in Table S2.

**Cells, Transfections, and Lytic Reactivation**

Human embryonic kidney (HEK) 293T cells were maintained in Dulbecco’s modified Eagle’s medium (DMEM, Invitrogen) supplemented with 10% fetal bovine serum (FBS, Invitrogen). For knockdown experiments, 293T cells were transfected twice with a final concentration of 70 nM siRNA target specific or DS scrambled negative control oligos (IDT), or 1 µg/ml of shRNA constructs (kindly provided by O. Mühlemann) using Lipofectamine 2000 (Invitrogen) at 96 and 48 hours prior to DNA transfection. Sequences of the siRNA oligos and shRNAs are listed in Table S4. For DNA transfections, reporter constructs (0.1 or 0.2 µg/ml) were transfected either alone or in combination with 0.2 µg/ml of the indicated SOX construct, using Effectene reagent (Qiagen) or Lipofectamine 2000 following the manufacturers’ protocol. Where specified, the Dcp2 E148Q dominant negative construct was also transfected at 0.2 µg/ml. Protein and RNA samples were collected 16-24 hours following DNA transfection as described below.

The KSHV-positive B cell line TREx BCBL1-RTA [11] (kindly provided by J. Jung and I. Mohr) was maintained in RPMI 1640 medium (Invitrogen) supplemented with 10% FBS, 200 µM L-glutamine (Invitrogen), 100 U/ml penicillin (Invitrogen), 100 µg/ml streptomycin (Invitrogen), and 50 µg/ml hygromycin B (Omega Scientific). To induce lytic reactivation of KSHV, TREx BCBL1-RTA cells were split to 2×10^5^ cells/ml and, 24 h later, induced with 20 ng/ml 2-*O*-tetradecanoylphorbol-13-acetate (TPA, Sigma), 1 µg/ml doxycycline (BD Biosciences), 500 ng/ml ionomycin (Fisher Scientific) [12].

To obtain pure fractions of GFP-SOX or GFP-expressing cells for the analysis of endogenous transcripts, HEK 293T cells transfected with pCDNA3.1-GFP-SOX [3] or pd2eGFP-N1 and were sorted on a Dako-Cytomation MoFlo High Speed Sorter at the Cancer Research Laboratory Flow Cytometry Facility at the University of California, Berkeley.

**Polysome Profiling**

Polysome profile analysis on samples were carried out using a procedure described in Jackson and Larkins [13] with minor modifications. 24 h post transfection or induction, cells were treated with 100 mg/ml cycloheximide for 30 minutes prior to harvesting in polysome extraction buffer [100 mM Tris-CL (pH8.0), 20 mM MgCl_2_, 200 mM KCl, 5 mM EGTA, 1 mM DTT, 1% (v/v) Triton X-100, 100 mg/ml cycloheximide, protease inhibitors cocktail (Roche)]. The soluble portion of the cell lysate was overlayed onto 15-60% sucrose gradients in gradient buffer [40mM Tris-CL (pH 7.4), 10 mM MgCl_2_, 20 mM KCl, 100 mg/ml cycloheximide], and centrifuged at 37,000 rpm in a SW41 rotor at 4°C for 100 min. Where indicated, extracts were pelleted through 60% sucrose using a Ti 60 rotor before layering on the gradient. Resolution of the RNP/40S fractions was carried out as described above, but using a 5%-20% gradient of sucrose and centrifuging for 120 min. One ml fractions were collected and analyzed using an ISCO model D density gradient fractionator attached to an ISCO model UA5 absorbance monitor at 254 nm absorbance. For Western blots, 50-100 µl of each fraction was used directly for SDS-PAGE. For Northern blots, total RNA was isolated from 500 µl of each fraction.

**DNase Assays**

DNase assays were carried out as previously described [14], with minor modifications. Proteins analyzed were in vitro transcribed (mMessage machine, Ambion) and translated using the Rabbit Reticulocyte Lysate system (Promega) in the presence of ^35^S-methionine. DNase activity was tested by incubation with 200 ng of linearized DNA in a total volume of 50 µl of degradation assay buffer (0.1 M MgCl_2_, 0.5 M Tris, pH 9.0, 100 µg/ml bovine serum albumin, 5 mM β-mercaptoethanol) at 37ºC for 10 minutes. DNA was then phenol/chloroform extracted and visualized by agarose gel electrophoresis. Protein expression was assessed by SDS-PAGE, and autoradiography of fixed gels.

**5’ Rapid amplification of cDNA ends (RACE)**

To map the sequences at the 5’ end of the degradation fragments, the First Choice RNA ligation-mediated rapid amplification of cDNA ends kit (First Choice RLM-RACE, Ambion) was used according to the manufacturer’s protocol with modifications. Briefly, an RNA adapter was ligated directly to total RNA, omitting the dephosphorylation and decapping steps used to select for capped messages, thus isolating only RNAs that already had a 5’ monophosphate as a result of RNase activity within the cells. After cDNA synthesis, PCR was carried out with nested adapter-specific primers and gene-specific primers (listed in Table S3). To enrich for intermediates of SOX-mediated decay, the dominant bands corresponding to the intermediates were excised, cloned and sequenced.

Table S1: QuikChange primers

| **Construct** | **Primer sequence** |
| --- | --- |
| dsRed2-100stop | CGCGTGATGAACTTCTAGGACGGCGGCGTGG |
| SOX W135V | agtctgaaccccgtggtcgacgccctgcgagac |
| SOX R139I | ccccgtgtgggacgccctgatcgacggaattatatcttcatc |
| SOX S144I | TGTGGGACGCCCTGCGAGACGGAATTATAATCTCATCCAAGTTTCAC |
| SOX S146I | GCCACTAGTAGTCAGATCCTGAACCCCGTGTGGG |
| SOX E184A | ggctgcggtgcgcggaggtggtgaa |
| SOX S219A | GGAATATTTGGCGTGGCCCTGGATTTCGCGGCG |
| SOX D221A | GGCGTGTCGCTGGCTTTCGCGGCGAAC |
| SOX D221S | GAATATTTGGCGTGTCGCTGAGCTTCGCGGCGAACGTCAAAAC |
| SOX E244A | GACCCTAACTGTAAAGTGTATGCAATAAAATGCAGGTTCAAGTAC |
| SOX K246I | AGTTTGACCCTAACTGTAAAGTGTATGAAATAATCTGCAGGTTCAAGTACAC |
| SOX Q376G | cgtgaacgtccgtcacagctacttttatggagtcttgctgcagagttcg |
| GFP- ΔTGAAG | TACGGCAAGCTGACCTTCATCTGCACCACC |
| GFP + nt.131-138 | GGACGAGCTGTACACCCTGAAGAGAAGCTTAGCC |

**Table S**2: Cloning primers

| **Construct** | **Primer sequence** |
| --- | --- |
| GFP/ DsRed2 SLII 5’ | F: ggcggatccagaaagtcaggccgggaagttcc  R: ggcggatccacccagtcctcctggggttgagtcg |
| GFP SLII coding | F: ggcTGTACAagaaagtcaggccgggaagttcc  R: ggcTGTACAacccagtcctcctggggttgagtcg |
| GFP/ DsRed2/ β-globin SLII 3’ | F: ggcGCGGCCGCagaaagtcaggccgggaagttcc  R: ggcGCGGCCGCacccagtcctcctggggttgagtcg |
| GFPrep | F: CCGTGTACAGCATGGTGAGCAAGGGCGAGGAGC  R: CCGTGTACAGGTAGGTCAGGGTGGTCACGAGGG |
| DsRed2 SLII coding | F: CGTGAAGCACCCCGCCGACATCaagaaagtcaggccgggaagttcc  R: GGGGAAGGACAGCTTCTTGTAGTCGGGcaacccagtcctcctggggttgagtc |
| GFP + nt 126-150 | F: GGCTGTACACGCTGACCCTGAAGTTC  R: GGCTGTACAGGGTGCAGATGAACTTCAGG |
| Pol I GFP | F: GCGTCTCATATTATGGTGAGCAAGGGCGAG  R: GCGTCTCAGGGGCTACACATTGATCCTAGCAGAAGCA |
| Pol III GFP | F: AGTCGATGCATATGGTGAGCAAGGGCGAG  R: AGTCAGATCTCTACACATTGATCCTAGCAGAAGCA |
| pBMN-SOX | F: CGGAATTCatggaggccacccccaca  R: actGTCGACctacgggctgtgagggacg |

Table S3: 5’ RACE primers

| **Construct** | **Primer sequence** |
| --- | --- |
| GFP | Outer: CCTCCTTGAAGTCGATGCCCTTC  Inner: CTCGATGCGGTTCACCAGGGTGTC |
| DsRed2 | Outer: TGTAACCATTATAAGCTGCAA  Inner: ATGATCTAGAGTCGCGGCCGCTAC |
| β-globin | Outer: CACCAGCCACCACTTTCTGATAGG  Inner: GTGACAAGCTGCACGTGGATCCTG |

**Table S4: siRNAs and shRNAs**

| **siRNA** | **Sequence (Sense strand of duplex)** | **Reference (if used previously)** |
| --- | --- | --- |
| hDcp1A * | #1 CCAGGAUCCUGAAGUAUUUGUGCAG  #2 CGAUCAUUGUGUUAAGUUAUGUGGG | N/A |
| hDcp2 | GGACUGGCUUUCUCGAAGAUUUGGC | [15] |
| hXrn1 | AGAUGAACUUACCGUAGAAAAUGTA | [15] |
| hRrp41 | CUAGUGAACUGUCAAUAUAGUUCAG | N/A |
| **shRNA** | **Target sequence** |  |
| pSUPuro-Xrn1 | AGAUGAACUUACCGUAGAA | [16] |

*oligos used as a mixture

**SUPPLEMENTAL REFERENCES**

1. Glaunsinger B, Ganem D (2004) Lytic KSHV infection inhibits host gene expression by accelerating global mRNA turnover. Mol Cell 13: 713-723.

2. Lee YJ, Glaunsinger BA (2009) Aberrant herpesvirus-induced polyadenylation correlates with cellular messenger RNA destruction. PLoS Biol 7: e1000107.

3. Clyde K, Glaunsinger B (2011) Deep sequencing reveals direct targets of gammaherpesvirus-induced mRNA decay and suggests that multiple mechanisms govern cellular transcript escape. PLoS One 6: e19655.

4. Singh G, Rebbapragada I, Lykke-Andersen J (2008) A competition between stimulators and antagonists of Upf complex recruitment governs human nonsense-mediated mRNA decay. PLoS Biol 6: e111.

5. Lykke-Andersen J (2002) Identification of a human decapping complex associated with hUpf proteins in nonsense-mediated decay. Mol Cell Biol 22: 8114-8121.

6. Johannes G, Carter MS, Eisen MB, Brown PO, Sarnow P (1999) Identification of eukaryotic mRNAs that are translated at reduced cap binding complex eIF4F concentrations using a cDNA microarray. Proc Natl Acad Sci U S A 96: 13118-13123.

7. Neumann G, Watanabe T, Ito H, Watanabe S, Goto H, et al. (1999) Generation of influenza A viruses entirely from cloned cDNAs. Proc Natl Acad Sci U S A 96: 9345-9350.

8. Hogg JR, Collins K (2007) Human Y5 RNA specializes a Ro ribonucleoprotein for 5S ribosomal RNA quality control. Genes Dev 21: 3067-3072.

9. Pijlman GP, Funk A, Kondratieva N, Leung J, Torres S, et al. (2008) A highly structured, nuclease-resistant, noncoding RNA produced by flaviviruses is required for pathogenicity. Cell Host Microbe 4: 579-591.

10. Geiser M, Cebe R, Drewello D, Schmitz R (2001) Integration of PCR fragments at any specific site within cloning vectors without the use of restriction enzymes and DNA ligase. BioTechniques 31: 88-90, 92.

11. Nakamura H, Lu M, Gwack Y, Souvlis J, Zeichner SL, et al. (2003) Global changes in Kaposi's sarcoma-associated virus gene expression patterns following expression of a tetracycline-inducible Rta transactivator. J Virol 77: 4205-4220.

12. Arias C, Walsh D, Harbell J, Wilson AC, Mohr I (2009) Activation of host translational control pathways by a viral developmental switch. PLoS Pathog 5: e1000334.

13. Jackson AO, Larkins BA (1976) Influence of Ionic Strength, pH, and Chelation of Divalent Metals on Isolation of Polyribosomes from Tobacco Leaves. Plant Physiol 57: 5-10.

14. Covarrubias S, Richner JM, Clyde K, Lee YJ, Glaunsinger BA (2009) Host shutoff is a conserved phenotype of gammaherpesvirus infection and is orchestrated exclusively from the cytoplasm. J Virol 83: 9554-9566.

15. Damgaard CK, Kahns S, Lykke-Andersen S, Nielsen AL, Jensen TH, et al. (2008) A 5' splice site enhances the recruitment of basal transcription initiation factors in vivo. Mol Cell 29: 271-278.

16. Eberle AB, Lykke-Andersen S, Muhlemann O, Jensen TH (2009) SMG6 promotes endonucleolytic cleavage of nonsense mRNA in human cells. Nat Struct Mol Biol 16: 49-55.
